# Association of Genetic and Phenotypic Assessments With Onset of Disordered Eating Behaviors and Comorbid Mental Health Problems Among Adolescents

**DOI:** 10.1001/jamanetworkopen.2020.26874

**Published:** 2020-12-02

**Authors:** Lauren Robinson, Zuo Zhang, Tianye Jia, Marina Bobou, Anna Roach, Iain Campbell, Madeleine Irish, Erin Burke Quinlan, Nicole Tay, Edward D. Barker, Tobias Banaschewski, Arun L. W. Bokde, Antoine Grigis, Hugh Garavan, Andreas Heinz, Bernd Ittermann, Jean-Luc Martinot, Argyris Stringaris, Jani Penttilä, Betteke van Noort, Yvonne Grimmer, Marie-Laure Paillère Martinot, Corinna Insensee, Andreas Becker, Frauke Nees, Dimitri Papadopoulos Orfanos, Tomáš Paus, Luise Poustka, Sarah Hohmann, Juliane H. Fröhner, Michael N. Smolka, Henrik Walter, Robert Whelan, Gunter Schumann, Ulrike Schmidt, Sylvane Desrivières

**Affiliations:** 1 Section of Eating Disorders, Department of Psychological Medicine, King’s College London, London, United Kingdom; 2 Centre for Population Neuroscience and Precision Medicine, Institute of Psychiatry, Psychology & Neuroscience, SGDP Centre, King’s College London, London, United Kingdom; 3 Institute of Science and Technology for Brain-Inspired Intelligence, Fudan University, Shanghai, China; 4 Ministry of Education-Key Laboratory of Computational Neuroscience and Brain-Inspired Intelligence, Fudan University, Shanghai, China; 5 Developmental Psychopathology Lab, Department of Psychology, King’s College London, London, United Kingdom; 6 Department of Child and Adolescent Psychiatry and Psychotherapy, Central Institute of Mental Health, Medical Faculty Mannheim, Heidelberg University, Mannheim, Germany; 7 Discipline of Psychiatry, Trinity College Institute of Neuroscience, Trinity College Dublin School of Medicine, Dublin, Ireland; 8 NeuroSpin, CEA, Université Paris-Saclay, F-91191 Gif-sur-Yvette, France; 9 Departments of Psychiatry and Psychology, University of Vermont, Burlington; 10 Charité–Universitätsmedizin Berlin, Corporate member of Freie Universität Berlin, Humboldt–Universität zu Berlin, and Berlin Institute of Health, Department of Psychiatry and Psychotherapy, Berlin, Germany; 11 Physikalisch-Technische Bundesanstalt, Braunschweig and Berlin, Germany; 12 INSERM U A10 “Developmental Trajectories & Psychiatry,” Université Paris-Saclay, Ecole Normale Supérieure Paris-Saclay, CNRS, Centre Borelli, Gif-sur-Yvette, France; 13 National Institute of Mental Health, National Institutes of Health, Bethesda, Maryland; 14 Department of Social and Health Care, Psychosocial Services Adolescent Outpatient Clinic Kauppakatu 14, Lahti, Finland; 15 MSB Medical School Berlin, Berlin, Germany; 16 Department of Child and Adolescent Psychiatry and Psychotherapy, Central Institute of Mental Health, Medical Faculty Mannheim, Heidelberg University, Mannheim, Germany; 17 Assistance Publique-Hôpitaux de Paris, Department of Child and Adolescent Psychiatry, Pitié-Salpêtrière Hospital, Paris France; 18 Department of Child and Adolescent Psychiatry and Psychotherapy, University Medical Centre Göttingen, Göttingen, Germany; 19 Institute of Medical Psychology and Medical Sociology, University Medical Center Schleswig Holstein, Kiel University, Kiel, Germany; 20 Bloorview Research Institute, Holland Bloorview Kids Rehabilitation Hospital and Departments of Psychology and Psychiatry, University of Toronto, Toronto, Ontario, Canada; 21 Technische Universität Dresden, Faculty of Medicine Carl Gustav Carus, Department of Psychiatry and Psychotherapy, Section of Systems Neuroscience, Dresden, Germany; 22 Deptartment of Psychiatry and Psychotherapy, Campus Charite Mitte, Humboldt University, Berlin, Germany and Institute for Science and Technology of Brain-inspired Intelligence (ISTBI), Fudan University, Shanghai, China; 23 Global Brain Health Institute, Trinity College Dublin School of Psychology, Dublin, Ireland; 24 The Eating Disorders Service, Maudsley Hospital, South London and Maudsley NHS Foundation Trust, London, United Kingdom

## Abstract

**Importance:**

Eating disorders are serious mental disorders with increasing prevalence. Without early identification and treatment, eating disorders may run a long-term course.

**Objective:**

To characterize any associations among disordered eating behaviors (DEBs) and other mental health disorders and to identify early associations with the development of symptoms over time.

**Design, Setting, and Participants:**

This multicenter, population-based, longitudinal cohort study used data from baseline (collected in 2010), follow-up 1 (collected in 2012), and follow-up 2 (collected in 2015) of the IMAGEN Study, which included adolescents recruited from 8 European sites. The present study assessed data from 1623 healthy adolescents, aged 14 years at baseline, recruited from high schools. Data analyses were performed from January 2018 to September 2019.

**Main Outcomes and Measures:**

Body mass index (BMI), mental health symptoms, substance use behaviors, and personality variables were investigated as time-varying associations of DEBs (dieting, binge eating, and purging) or change in BMI over time. Polygenic risk scores were calculated to investigate genetic contributions associated with BMI, attention-deficit/hyperactivity disorder (ADHD) and neuroticism to DEBs.

**Results:**

In this cohort study of 1623 adolescents (829 girls [51.1%]) recruited at a mean (SD) age of 14.5 (0.4) years and followed up at ages 16 and 19 years, 278 adolescents (17.1%) reported binge eating, 334 adolescents (20.6%) reported purging, and 356 adolescents (21.9%) reported dieting at 14, 16, or 19 years. Among the precursors of DEBs, high BMI was associated with future dieting (OR, 3.44; 95% CI, 2.09-5.65). High levels of neuroticism (OR, 1.04; 95% CI, 1.01-1.06), conduct problems (OR, 1.41; 95% CI, 1.17-1.69), and deliberate self-harm (OR, 2.18; 95% CI, 1.37-3.45) were associated with future binge eating. Low agreeableness (OR, 0.95; 95% CI, 0.92-0.97), deliberate self-harm (OR, 2.59; 95% CI, 1.69-3.95), conduct problems (OR, 1.42; 95% CI, 1.20-1.68), alcohol misuse (OR, 1.31; 95% CI, 1.10-1.54), and drug abuse (OR, 2.91; 95% CI, 1.78-4.74) were associated with future purging. Polygenetic risk scores for BMI were associated with dieting (at 14 years: OR, 1.27; lower bound 95% CI, 1.08; at 16 years: OR, 1.38; lower bound 95% CI, 1.17); ADHD, with purging (at 16 years: OR, 1.25; lower bound 95% CI, 1.08; at 19 years, OR, 1.23; lower bound 95% CI, 1.06); and neuroticism, with binge eating (at 14 years: OR, 1.32; lower bound 95% CI, 1.11; at 16 years: OR, 1.24; lower bound 95% CI, 1.06), highlighting distinct etiologic overlaps between these traits. The DEBs predated other mental health problems, with dieting at 14 years associated with future symptoms of depression (OR, 2.53; 95% CI, 1.56-4.10), generalized anxiety (OR, 2.27; 95% CI, 1.14-4.51), deliberate self-harm (OR, 2.10; 95% CI, 1.51-4.24), emotional problems (OR, 1.24; 95% CI, 1.08-1.43), and smoking (OR, 2.16; 95% CI, 1.36-3.48). Purging at 14 years was also associated with future depression (OR, 2.87; 95% CI, 1.69-5.01) and anxiety (OR, 2.48; 95% CI, 1.49-4.12) symptoms.

**Conclusions and Relevance:**

The findings of this study delineate temporal associations and shared etiologies among DEBs and other mental health disorders and emphasize the potential of genetic and phenotypical assessments of obesity, behavioral disorders, and neuroticism to improve early and differential diagnosis of eating disorders.

## Key Points

**Question:**

Do associations exist between disordered eating behaviors and other mental health disorders in adolescence, and if so, to what extent are those associations genetically predisposed?

**Findings:**

Longitudinal assessments in this cohort study of a population-based sample of 1623 adolescents indicated that body mass index (BMI), neuroticism, impulse control, and addiction-related behaviors at 14 years of age were differentially associated with future disordered eating behaviors and symptoms of depression and generalized anxiety. Genetic analyses suggested etiologic overlaps between BMI, neuroticism, and attention-deficit/hyperactivity disorder with dieting, binge eating, and purging, respectively.

**Meaning:**

Genetic and phenotypic assessments of BMI, impulse control problems, and personality may inform early and differential diagnoses of eating disorders.

## Teaser

This cohort study across numerous European sites assesses whether associations and shared etiologies exist between disordered eating behaviors (dieting, purging, and binge eating) and other mental health disorders in early adolescence.

## Introduction

Eating disorders (EDs) are common, disabling, and deadly psychiatric disorders that affect up to 15% of young women and up to 3% of young men in middle- to high-income countries.^1,2^ Peak onset is between 16 and 19 years of age ^3,4^ a developmentally sensitive time. Main ED diagnoses include anorexia nervosa (AN), bulimia nervosa (BN), binge eating disorder and other specified feeding and eating disorders, all characterized by disturbed eating or compensatory purging behaviors, such as vomiting or misuse of laxatives or diuretics, for the purpose of weight control. Prior to the onset of full-syndrome EDs, people typically display milder or more sporadic subclinical disordered eating behaviors (DEBs), such as restrictive eating, binge eating, or purging.^5^ Identifying risk factors and correlates of these DEBs is likely to be an effective way to facilitate early detection and development of early targeted interventions to prevent disease progression.

Eating disorders are highly comorbid with other psychiatric disorders, notably anxiety,^6,7,8,9^ mood,^10^ and impulse-control disorders, such as attention-deficit/hyperactivity disorder (ADHD).^11^ The latter seems to be more associated with binge/purge spectrum EDs^12^ or obesity,^13^ than with AN.^12,14^ However, few studies have prospectively determined the extent to which these co-occurring psychiatric disorders predispose to, precipitate, coincide with, or are a consequence of an ED.^15^ In addition, personality disorders and relevant traits, notably neuroticism, have been consistently associated with ED symptomatology.^13^

The etiology of EDs is complex,^16^ with evidence for overlapping and distinct biopsychosocial factors implicated in risk for different clinical EDs or DEBs.^17,18,19^ Widely accepted risk factors include sex and body mass index (BMI),^7,8,20,21^ and sociocultural factors.^22^

Twin studies indicate that genetic factors account for up to 74% of the variance in liability to AN^23^ and between 55% and 83% of the variance in the liability to BN.^24^ Shared genetic factors also partly account for diagnostic transitions between AN and BN^25^ and common comorbidities.^26^ The DEBs themselves appear to be moderately heritable; binge eating, self-induced vomiting, and dietary restraint are estimated to be between 44% and 72% heritable.^27,28^

Recent reports considering biopsychosocial aspects of EDs suggest that environmental and psychological factors interact with and influence the expression of genetic risk to cause eating pathology.^16,20,21^ Consequently, progress in understanding etiologic and pathophysiologic mechanisms implicated in DEBs and EDs is likely to be gained from a multimodal approach integrating both genetic and longitudinal measures into biobehavioral models.

The present study uses the longitudinal and multimodal IMAGEN cohort of approximately 1600 adolescents to characterize the development of DEBs across adolescence and their temporal associations with other psychopathologies. We combined genetic and phenotypic data to analyze traits associated with ED psychopathology in adolescents. We thereby identify early factors and pathophysiologic mechanisms associated with the development of these behaviors and comorbid mental disorders.

## Methods

### Participants

We used data from IMAGEN, a multicenter prospective longitudinal population study of adolescents aged 14 years aimed at identifying the genetic and neurobiological basis of individual variability in quantitative neuropsychological traits and determining their value for estimating the development of frequent psychiatric disorders. Full details of the IMAGEN project can be found on their website and in the eAppendix in the Supplement. Adolescents were recruited from high schools at 8 European sites when aged 14 years (baseline)^29^ and followed up at 16 years (follow-up 1, collected in 2012) and 19 years (follow-up 2, collected in 2015). This work is reported following the Strengthening the Reporting of Observational Studies in Epidemiology (STROBE) reporting guideline for cohort studies. Each site received approval from their local research ethics committee, and written consent or assent was obtained from each participant in a manner consistent with the Declaration of Helsinki.^30^ Participants received compensation of £100 (approximately $130) or €100 (approximately $118) in either vouchers or bank transfers, depending on site and follow-up assessment.

The final sample for this study included 1623 adolescents who reported any DEB at baseline, follow-up 1, or follow-up 2 or who screened negative for the Development and Well-Being Assessment (DAWBA) section P screening eating disorder questions at each corresponding age. Individuals with missing data on sex (5.54%), study site (14.62%), DEBs, mental health disorder symptoms, or personality traits (10.22%) were excluded from the final analyses. Only individuals with complete cases were included in the analyses.

### Assessments of ED Psychopathology

#### DEB Variables

The DEB variables were derived from adolescents self-report on DAWBA section P (Eating Behaviors).^31^ The ED module of the DAWBA was formed by structured questions assessing the eating patterns of the participants.^32^ Binary variables for DEBs, including binge eating, purging, and dieting, were derived from responses to the DAWBA (eAppendix in the Supplement). Healthy controls were defined as adolescents whose results on DAWBA section P screening questions at each corresponding age were negative. These individuals are referred to hereinafter as having no DEBs.

#### DEB Development

We categorized into groups participants who developed symptoms over time for each of the 3 DEBs. Accordingly, *developers* were defined as individuals who reported a given DEB at ages 16 or 19 years, but not at age 14 years. These groups were compared with individuals who reported no DEBs at all ages.

We also assessed participant distress about eating pattern, body shape, and weight with the DAWBA and attitudinal aspects of ED (restraint, eating concern, weight concern, and shape concern) using the Eating Disorder examination questionnaire (eAppendix in the Supplement).

### Assessment of Mental Health Disorder Symptoms and Personality Traits

#### Mental Health Disorder Symptoms

The DAWBA computer-based estimations^33^ from self-reports were used to estimate the probability of currently having or likelihood of developing over time a mental health disorder. Diagnoses investigated in this study were included if more than 5% of the IMAGEN sample scored higher than 0 or 0.01% for the DAWBA computer estimation. We defined developers as described above (eAppendix in the Supplement).

#### Alcohol and Substance Use Behaviors

Alcohol and substance use behaviors were assessed using the European School Survey Project on Alcohol and Other Drugs^34^ and The Alcohol Use Disorders Identification Test.^35^

#### Emotional and Behavioral Problems

The 25 items of the Strengths and Difficulties Questionnaire, scored 0 for not true, 1 for somewhat true, and 2 for certainly true, were used to assess 5 subscales: emotional symptoms, conduct problems, hyperactivity/inattention, peer relationship problems, and prosocial behavior.^36^ Mean internal consistency (Cronbach α) across all subscales was 0.53.

#### Personality Traits

The 60-item Neuroticism-Extraversion Openness Five-Factor Inventory included the dimensions of extraversion, agreeableness, conscientiousness, neuroticism, and openness to experience.^37^ Items were scored on a 5-point scale anchored from 0 for strongly agree to 4 for strongly disagree. Mean Cronbach α across the 5 subscales was 0.55.

### Polygenic Risk Scores Analysis

Acquisition of genetic data and quality controls are detailed in the eAppendix in the Supplement. After quality control, 1975 cases and 502 single-nucleotide variations with alternative bases at nucleotide 160 were available for polygenetic risk score (PRS) analyses. Polygenic risk scores were generated for neuroticism, BMI, and ADHD symptoms based on summary statistics from large-scale genome-wide association studies on neuroticism,^38^ BMI calculated as weight in kilograms divided by height in meters squared,^39^ and ADHD measured by the hyperactivity/inattention subscale in the Strengths and Difficulties Questionnaire.^40^ We also generated PRSs for the 2 subcomponents of neuroticism: depressed affect and worry^38^ (eAppendix in the Supplement).

### Statistical Analysis

Generalized estimating equations^41,42^ using DEBs as time-varying estimators and logistic and linear regression models controlling for sex and study site were used to investigate the prospective associations, correlates, and outcomes of DEBs (eAppendix in the Supplement). Logistic regressions were used to identify traits and behaviors at age 14 years that were associated with the development of DEBs (ie, with DEB developer groups), focusing analyses on traits or behaviors found to be significantly associated with DEBs in the generalized estimating equation models. For each DEB group, individuals reporting a DEB at 14 years were excluded from these longitudinal analyses. All analyses were controlled for sex and site.

#### Associations Between and Future Mental Health Outcomes

Logistic regression models were used to investigate whether any of the 3 DEBs at baseline (aged 14 years) were associated with the development of mental health or substance use–related outcomes or emotional and behavioral problems. For categorical variables (ie, DAWBA diagnoses or drug use for the European School Survey Project on Alcohol and Other Drugs), “developer” groups characterized by absence of symptoms at 14 years and occurrence at 16 or 19 years were tested for associations with DEB at 14 years. In the case of scaled measures (ie, the Strengths and Difficulties Questionnaire subscales and The Alcohol Use Disorders Identification Test total score), the mean values for ages 16 and 19 years were calculated and use to test for associations with DEBs, controlling for symptoms at14 years. Sex and study sites were included as covariates.

#### PRS Analyses

Logistic regression models were performed to test associations between PRSs and DEBs and mediation models to investigate whether related phenotypes mediated the association between PRS and DEBs at 14 years. Continuous variables were transformed to *z* scores, and analyses controlled for sex, study site, and population stratification (the first 4 multidimensional scaling components of the genotype data). Confidence intervals for the indirect effect were estimated with 5000 bootstrap samples by using the PROCESS macro, version 3.2,^43^ in SPSS, version 25 (IBM Corporation). All tests were corrected for multiple testing using the Benjamin-Hochberg procedure with a false discovery rate lower than 0.05,^44^ using the formula *P* value × (total number of hypotheses tested)/(rank of the *P* value). The PRS analyses were corrected for multiple testing using the Bonferroni procedure^45^ with 1-tailed tests for statistical significance. All data analyses were conducted from January 2018 to September 2019.

## Results

The study comprised 1623 adolescents of European descent (829 girls [51.1%]) recruited at a mean (SD) age of 14.5 (0.4) years and followed up at the mean (SD) ages of 16.5 (0.7) years and 19.4 (1.5) years for which information on DEB was available at any age. Of these participants, 1509 had DEB information at 14 years, 1317 at 16 years, and 853 at 19 years. Lifetime occurrences of DEBs were 278 (17.1%) for binge eating, 334 (20.6%) for purging, and 356 (21.9%) for dieting, and 1623 individuals reporting DEBs or “no DEBs” at baseline, follow-up 1, or follow-up 2 were included in this study. As expected, DEBs were more common in girls (range, 456-452 [77%-88%]) and were associated with significant distress (range, 451-516 [76%-87%] reporting distress; *P* < .05). The mean BMI (SD) at 14 years of age was 20.9 (3.4), with higher BMI associated with all DEBs (*P* < .001) and with no differences between DEB groups (eTable 1 in the Supplement). For developers (ie, individuals reporting a DEB at 16 or 19 years but not at 14 years), 127 (13.6%) were classified as binge-eating developers, 207 (16.8%) as purge developers, and 86 (17.1%) as dieting developers. The DEB developers also developed specific concerns with eating and body shape (eTable 2 in the Supplement).

### DEBs and Their Development Across Adolescence

We dissected the development of disordered eating patterns during adolescence by comparing individuals who did not report DEB at 14 years but developed DEBs at 16 or 19 years (developers) with individuals who did not endorse these behaviors at any time (healthy controls). Between 86 and 207 individuals (3.5%-8.4%) who reported no DEB at 14 years reported DEBs at 16 or 19 years (eTable 2 in the Supplement). Future purging was associated with future dieting (odds ratio [OR], 39.07; 95% CI, 18.14 -84.15) such that of the individuals who developed dieting behavior at 16 or 19 years, 33 of 86 (38.4%) also developed purging. Similarly, 33 of 207 individuals who purged in the future (15.7%) also dieted in the future. Engaging in future binge eating was also associated with engaging in future dieting (OR, 40.53; 95% CI, 16.203-100.41). Of the individuals who engaged in binge eating in the future, 25 of 167 (14.9%) also dieted in the future. Conversely, 25 of 86 individuals (35.7%) who dieted in the future also binge ate. The development of purging was also associated with the development of binge eating (OR, 6.58; 95% CI, 4.18-9.83) such that 61 of 207 individuals (29.9%) who developed purging behavior at age 16 or 19 also developed binge eating behavior. Similarly, 62 of 167 individuals (37.1%) who engaged in future binge eating also developed purging behavior.

**Figure 1.**
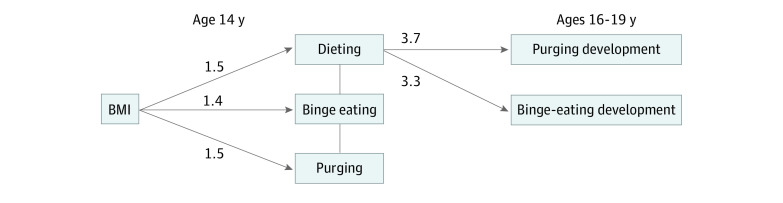
Significant Associations Between Body Mass Index (BMI), Disordered Eating Behaviors (DEBs) at 14 Years, and the Development of Future DEBs at 16 or 19 Years All models account for both sex and study site. For each DEB shown in the middle column, other DEBs have been accounted for in the model (eg, in the association between dieting and purging development, binge eating at baseline was controlled for). The arrows indicate a statistically significant association with the false discovery rate correction (corrected *P* < .05 for 15 tests); numbers next to arrows, odds ratios.

Dieting at 14 years was associated with both future binge eating (OR, 3.26; 95% CI, 1.71-6.18) and future purging (OR, 3.67; 95% CI, 2.02-6.67), when controlling for purging, binge eating, and BMI at baseline. When the role of binge eating, dieting, and BMI at baseline were accounted for, binge eating at age 14 was not significantly associated with future purging (OR, 3.35; 95% CI, 1.16-9.66); purging at 14 years was not significantly associated with future binge eating (OR, 1.64; 95% CI, 0.51-5.19); binge eating at 14 years was not significantly associated with future dieting (OR, 2.93; 95% CI, 0.52-16.42); and purging at 14 years was not significantly associated with future dieting (OR, 2.26; 95% CI, 0.35-15.78 (Table 1 and Figure 1). Body mass index was specifically associated with future dieting, even when the other DEBs were controlled for in the model (OR, 3.44; 95% CI, 2.09-5.65), whereas future binge-eating (OR, 1.48; 95% CI, 0.81-2.74) and purging (OR, 1.21; 95% CI, 0.72-2.01) were not (Table 1 and Figure 1).

**Table 1.** Behavioral and Psychopathologic Factors at 14 Years Estimating Development of DEB at 16 or 19 Years^a^

Factor	Future binge eating (n = 167)		Future purging (n = 207)		Future dieting (n = 81)	
	OR (95% CI)	*P* value	OR (95% CI)	*P* value	OR (95% CI)	*P* value
Aged 14 y						
BMI	1.46 (1.20-1.77)^d^	1.3 × 10^−4^^d^	1.45 (1.21-1.75)^d^	6.10 × 10^−5^^d^	3.35 (2.36-4.74)^d^	9.80 × 10^−12^^d^
BMI^b^	1.48 (0.81-2.74)	.02	1.21 (0.72-2.01)	.47	3.44 (2.09-5.65)^d^	9.40 × 10^−12^^d^
Dieting^b^	3.26 (1.71-6.18)^d^	3.0 × 10^−4^^d^	3.67 (2.02-6.67)^d^	1.80 × 10^−5^^d^	Omitted^c^	Omitted^c^
Binge eating^b^	Omitted^c^	Omitted^c^	3.35 (1.16-9.66)	.02	2.93 (0.52-16.42)	.22
Purging^b^	1.64 (0.51-5.19)	.40	Omitted^c^	Omitted^c^	2.26 (0.35-15.78)	.41
DAWBA (aged 14 y)						
Depression	1.44 (0.87-2.37)	1.50 × 10^−1^	1.42 (0.89-2.25)	1.37 × 10^−1^	1.16 (0.51-2.63)	7.10 × 10^−1^
Generalized anxiety	1.35 (0.66-2.79)	4.06 × 10^−1^	1.93 (1.01-3.72)	4.80 × 10^−2^	2.23 (0.65-7.64)	2.00 × 10^−1^
Self-harm	2.18 (1.37-3.45)^d^	8.70 × 10^−4^^d^	2.59 (1.69-3.95)^d^	9.90 × 10^−6^^d^	1.23 (0.52-2.92)	6.24 × 10^−1^
OCD	1.31 (0.97-1.78)	8.20 × 10^−2^	Omitted^c^	Omitted^c^	1.56 (1.01-2.41)	4.30 × 10^−2^
Social phobia	0.87 (0.32-2.36)	7.94 × 10^−1^	Omitted^c^	Omitted^c^	0.44 (0.05-3.51)	4.38 × 10^−1^
Panic disorder	2.27 (0.56-9.24)	.25	1.05 (0.72-1.54)	7.84 × 10^−1^	Omitted^c^	Omitted^c^
PTSD	1.59 (1.09-2.32)	1.40 × 10^−2^	1.46	5.60 × 10^−2^	1.59 (0.86-2.96)	1.35 × 10^−1^
Conduct disorder	2.36 (1.17-4.77)	1.60 × 10^−2^	2.73 (1.47-5.09)^d^	1.50 × 10^−3^^d^	1.16 (0.38-3.49)	7.96 × 10^−1^
ADHD	Omitted^c^	Omitted^c^	1.71 (0.83-3.47)	1.41 × 10^−1^	Omitted^c^	Omitted^c^
Oppositional defiant (*ICD-10*)	2.52 (1.23-5.14)	1.10 × 10^−2^	1.11 (0.52-2.38)	7.75 × 10^−1^	Omitted^c^	Omitted^c^
SDQ (aged 14 y)						
Emotional problems	1.24 (1.03-1.48)	1.80 × 10^−2^	1.21 (1.03-1.42)	1.90 × 10^−2^	1.15 (0.89-1.49)	2.63 × 10^−1^
Conduct problems	1.41 (1.17-1.69)^d^	2.50 × 10^−4^^d^	1.42 (1.20-1.68)^d^	4.10 × 10^−5^^d^	1.13 (0.87-1.46)	3.41 × 10^−1^
Hyperactivity/inattention	1.25 (1.04-1.50)	1.40 × 10^−2^	1.38 (1.17-1.63)^d^	9.50 × 10^−5^^d^	1.16 (0.92-1.48)	2.08 × 10^−1^
Peer problems	1.24 (1.03-1.48)	1.90 × 10^−2^	0.99 (0.83-1.17)	9.16 × 10^−1^	1.30 (1.03-1.64)	2.50 × 10^−2^
Prosocial	0.94 (0.77-1.15)	6.02 × 10^−1^	Omitted^c^	Omitted^c^	Omitted^c^	Omitted^c^
AUDIT (aged 14 y)						
Alcohol misuse	1.07 (0.88-1.31)	4.65 × 10^−1^	1.31 (1.10-1.54)^d^	1.90 × 10^−3^^d^	1.04 (0.78-1.39)	7.42 × 10^−1^
ESPAD (aged 14 y)						
Drug use (>once in the past year)	1.36 (0.73-2.53)	3.20 × 10^−1^	2.91 (1.78-4.74)^d^	2.00 × 10^−5^^d^	2.28 (1.06-4.87)	3.30 × 10^−2^
Smoking	1.17 (0.24-5.71)	8.38 × 10^−1^	2.57 (0.85-7.79)	9.50 × 10^−2^	Omitted^c^	Omitted^c^
Binge drinking	Omitted^c^	Omitted^c^	1.19 (0.78-1.83)	4.26 × 10^−1^	Omitted^c^	Omitted^c^
NEO-FFI (aged 14 y)						
Neuroticism	1.04 (1.01-1.06)^d^	3.00 × 10^−3^^d^	1.01 (0.99-1.04)	1.20 × 10^−1^	1.04 (1.01-1.08)	2.20 × 10^−2^
Extraversion	0.96 (0.94-0.99)	4.20 × 10^−2^	1.01 (0.98-1.03)	5.10 × 10^−1^	0.97 (0.93-1.02)	3.30 × 10^−1^
Agreeableness	0.96 (0.93-0.99)	1.10 × 10^−2^	0.95 (0.92-0.97)^d^	1.80 × 10^−4^^d^	0.95 (0.92-0.99)	3.90 × 10^−2^
Conscientiousness	Omitted^c^	Omitted^c^	0.97 (0.95-0.99)	1.60 × 10^−2^	Omitted^c^	Omitted^c^

Abbreviations: ADHD, attention-deficit/hyperactivity disorder; AUDIT, The Alcohol Use Disorders Identification Test; BMI, body mass index; DAWBA, Development and Well-being Assessment; DEB, disordered eating behaviors; ESPAD, The European School Survey Project on Alcohol and Other Drugs; *ICD-10*, *International Statistical Classification of Disease, Tenth Revision*; NEO-FFI, Neuroticism-Extraversion Openness Five-Factor Inventory; OCD, obsessive-compulsive disorder; OR, odds ratio; PTSD, posttraumatic stress disorder; SDQ, Strengths and Difficulties Questionnaire.

^a^ All models controlled for sex and study site as covariates.

^b^ The development of other DEBs have been controlled for as additional covariates in the analysis.

^c^ Analyses have been omitted based on nonsignificant results derived from generalized estimating equations models (eTables 3 and 4 in the Supplement).

^d^ Statistical significance with false discover rate correction (corrected *P* < .05 for 68 tests). Benjamin-Hochberg threshold for significance of *P* = .01.

### DEBs and Comorbid Psychopathology, Co-occurring Behaviors, and Personality Traits in Adolescence

We used generalized estimating equation models to investigate the associations between DEBs and comorbid psychopathology, behaviors, and personality from 14 to 19 years (eTable 4 in the Supplement). Comorbid psychopathology shared across all DEBs (binge eating, purging, and dieting) included depression, generalized anxiety, deliberate self-harm, posttraumatic stress disorder, and conduct disorder. By contrast, higher rates of obsessive-compulsive disorder and binge eating (OR, 4.41; 95% CI, 1.18-16.39) and dieting (OR, 12.15; 95% CI, 2.31- 64.03) and social phobia symptoms were associated with binge eating (OR, 4.48; 95% CI, 2.51- 8.01) and dieting (OR, 5.09; 95% CI, 2.31-11.17), whereas ADHD (OR, 4.03; 95% CI, 1.67-9.68) and oppositional defiant disorder behaviors (OR, 3.03; 95% CI, 1.42-6.46) were more likely associated with purging. Accordingly, both internalizing (emotional and peer relationship problems) and externalizing (hyperactivity/inattention and conduct problems) tended to be higher in individuals reporting binge eating, purging, and dieting throughout adolescence (eTable 4 in the Supplement). Addiction-related behaviors, such as alcohol use (OR, 1.28; 95% CI, 1.21-1.37), drug use (OR, 5.32; 95% CI, 3.48-8.12), and smoking (OR, 4.61; 95% CI, 2.82-7.54), were also higher in those reporting ED behaviors than in controls, with the likelihood for engaging in such behaviors being particularly high among individuals who purged. The DEBs were also characterized by distinct personality profiles, with high levels of neuroticism significantly associated with binge eating (OR, 2.37; 95% CI, 1.98-2.83), purging (OR, 2.08; 95% CI, 1.74-2.48) and dieting (OR, 2.47; 95% CI, 2.03-3.01), and low levels of agreeableness significantly associated with binge eating (OR, 0.61; 95% CI, 0.51-0.79), purging (OR, 0.65; 95% CI, 0.55-0.76) and dieting (OR, 0.65; 95% CI, 0.55-0.79).

### Psychopathologic Correlates and Risk Factors for DEBs

We then investigated which of these psychopathologic behaviors were risk factors (rather than correlates) of future DEBs. For each of the significant associations reported above, we tested whether the variable assessed at 14 years predated the onset of future DEBs (ie, was significantly associated with the DEBs, comparing control vs developer groups) (Table 1). Although BMI was specifically associated with dieting (see above), future binge eating and future purging were associated with shared and distinct sets of psychopathologic and personality profiles. Future binge eating (OR, 2.18; 95% CI, 1.37-3.45) and future purging (OR, 2.59; 95% CI, 1.69-3.95) were associated with self-harm as well as with conduct problems (future binge eating: OR, 1.41; 95% CI, 1.17-1.69; future purging: OR, 1.42; 95% CI, 1.20-1.68). Future binge eating was specifically associated with high levels of neuroticism at age 14 (OR, 1.04; 95% CI, 1.01-1.06) and future purging with a set of symptoms composed of conduct disorder (OR, 2.73; 95% CI, 1.47-5.09), drug use (OR, 2.91; 95% CI, 1.78-4.74), alcohol misuse (OR, 1.31; 95% CI, 1.10-1.54), and low agreeableness (OR, 0.95; 95% CI, 0.92-0.97).

Testing whether precursors for DEB development were also associated with overweight status, we found that low agreeableness (OR, 0.81; 95% CI, 0.72-0.92) and high conduct problems (OR, 1.32; 95% CI, 1.15-1.46) at age 14 both associated with overweight status at 14 years. In addition, conduct problems at 14 years were associated with future overweight status of individuals of normal weights at 14 years (OR, 1.31; 95% CI, 1.02-1.71).

### Polygenic Information on Psychopathologic Risk Factors and DEBs

The analyses presented showed that BMI, neuroticism, and ADHD symptoms were risk factors for developing different DEBs, predating the appearance of these DEBs. We further investigated whether polygenic information related to these risk factors was associated with the corresponding DEB at each age (eTable 5 in the Supplement). The BMI PRS, which explained 5.15% (*P* < .01) of the variance in BMI at 14 years, was associated with dieting (at 14 years: OR, 1.27; lower bound 95% CI, 1.08; at 16 years: OR, 1.38; lower bound 95% CI, 1.17) and purging (at 14 years: OR, 1.34; lower bound 95% CI, 1.12; at 16 years: OR, 1.32; lower bound 95% CI, 1.14). The ADHD PRS, which explained 1.11% of the variance of ADHD symptoms at 14 years, was associated with purging (at 16 years: OR, 1.25; lower bound 95% CI, 1.08; at 19 years: OR, 1.23; lower bound 95% CI, 1.06). Finally, the full-scale neuroticism PRS, which explained 1.64% of the variance in neuroticism at 14 years, was associated with binge eating (at 14 years: OR, 1.32; lower bound 95% CI, 1.11; at 16 years: OR, 1.24; lower bound 95% CI, 1.06). Testing for the specific contributions of the 2 neuroticism subcomponents (depressed affect and worry) on binge eating at 14 years indicated significant associations with the PRS for depressed affect, but not worry, at 14 years (OR, 1.34; lower bound 95% CI, 1.12) and at 16 years (OR, 1.29; lower bound 95% CI 1.09). Accordingly, the PRS associated with depressed affect explained 0.86% of the neuroticism variance at 14 years, and worry explained 0.17% of the neuroticism variance at 14 years.

Mediation analyses (eFigure 2 in the Supplement) indicated that BMI mediated the associations between BMI PRS and dieting (indirect effect, 0.23; 95% CI, 0.17-0.32; 83.2% mediated) and purging (indirect effect, 0.14; 95% CI, 0.08-0.20; 41.0% mediated) at 14 years. Neuroticism partially mediated the effects of the neuroticism PRS on binge eating at 14 years (indirect effect, 0.057; 95% CI, 0.04-0.11; 23.9% mediated). Conversely, we did not find evidence of mediation by ADHD symptoms on associations between ADHD PRS and purging at 16 (indirect effect, 0.034; 95% CI, −0.012 to 0.065). Overall, these findings highlighted distinct etiologic overlaps between BMI, neuroticism, and ADHD and DEBs.

### Association Between DEBs at 14 Years and Future Health-Related Outcomes

**Figure 2.**
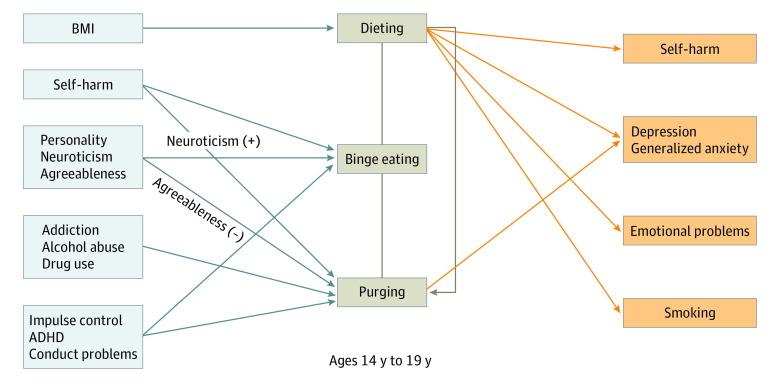
Time-Varying Associations and Outcomes of Disordered Eating Behaviors (DEBs) From 14 to 19 Years Associations between future DEBs (gray boxes) at age 14 and outcomes of DEBs at 16 or 19 years (orange boxes). Dieting at 14 years was associated with future binge eating and purging at 16 or 19 years, and binge eating at 14 years was associated with future purging at 16 or 19 years. The arrows indicate a significant association with the false discovery rate correction (corrected *P* < .05 for 89 tests). ADHD indicates attention-deficit/hyperactivity disorder; BMI, body mass index.

Finally, we considered the effects of DEBs on health-related outcomes by measuring associations between DEBs at 14 years and the later development of other mental health–related symptoms and change in BMI (Table 2, Figure 2). Dieting at 14 years was associated with future symptoms of depression (OR, 2.53; 95% CI, 1.56-4.10), generalized anxiety (OR, 2.27; 95% CI, 1.14-4.51), deliberate self-harm (OR, 2.10; 95% CI, 1.51-4.24), emotional problems (OR, 1.24; 95% CI, 1.08-1.43) and smoking (OR, 2.16; 95% CI, 1.36-3.48). Purging at 14 years was associated with future depression (OR, 2.87; 95% CI, 1.69-5.01) and anxiety (OR, 2.48; 95% CI, 1.49-4.12) symptoms. Binge eating was not significantly associated with future psychopathology.

**Table 2.** DEBs at 14 Years as Estimators of Future Health-Related Outcomes at 16 or 19 Years^a^

Outcome	Binge eating at 14 y (n = 107)		Purging at 14 y (n = 124)		Dieting at 14 y (n = 160)	
	OR (95% CI)	*P* value	OR (95% CI)	*P* value	OR (95% CI)	*P* value
BMI						
Change in BMI	1.01 (0.98-1.04)	.36	1.02 (0.99-1.07)	.24	0.99 (0.88-1.07)	.99
DAWBA						
Depression development	1.09 (0.59-2.03	.77	2.87 (1.69-5.01)^c^	3.40 × 10^−3^^c^	2.53 (1.56-4.10)^c^	2.80 × 10^−3^^c^
Generalized anxiety development	1.91 (0.76-2.51)	.02	2.48 (1.49-4.12)^c^	3.80 × 10^−3^^c^	2.27 (1.14-4.51)^c^	3.20 × 10^−4^^c^
Self-harm development	1.04 (0.53-2.06)	.91	2.03 (1.11-3.72)	.02	2.10 (1.51-4.24)^c^	3.40 × 10^−3^^c^
Social phobia development	1.17 (0.84-3.41)	.70	1.62 (0.79-3.14)	.18	0.54 (0.33-1.94)	.25
Panic disorder development	1.28 (0.51-4.48)	.69	2.43 (1.13-7.28)	.08	1.36 (0.32-4.08)	.63
Conduct disorder development	1.30 (0.65-3.07)	.58	1.62 0.92-3.81)	.28	1.12 (0.50-2.39)	.80
ADHD development	Omitted^b^	Omitted^b^	2.89 (0.89-6.56)	.06	Omitted^b^	Omitted^b^
Oppositional defiant (*ICD-10*) development	0.32 (0.11-1.89)	.27	1.99 (0.87-4.28)	.16	Omitted^b^	Omitted^b^
SDQ						
Emotional problems development	1.02 (0.87-1.20)	.76	1.21 (1.04-1.39)	.01	1.24 (1.08-1.43)^c^	2.20 × 10^−3^^c^
Conduct problems development	1.12 (0.46-2.42)	.38	1.06 (0.61-2.08)	.59	1.18 (0.97-1.48)	.15
Hyperactivity/inattention development	1.04 (0.87-1.24)	.62	1.09 (0.93-1.27)	.27	1.10 (0.94-1.28)	.21
Peer problems development	1.22 (0.95-1.57)	.11	1.33 (1.06-1.66)	.01	1.11 (0.89-1.39)	.33
Prosocial development	1.04 (0.79-1.38)	.75	Omitted^b^	Omitted^b^	Omitted^b^	Omitted^b^
AUDIT						
Alcohol misuse development	1.07 (0.98-1.17)	.12	1.06 (0.97-1.15)	.15	1.08 (1.00-1.17)	.65
ESPAD						
Drug use (>1 y) development	1.12 (0.52-2.45)	.73	1.02 (0.32-1.78)	.94	1.66 (0.61-2.47)	.04
Smoking development	1.85 (1.06-3.18)	.03	1.34 (0.78-2.34)	.28	2.16 (1.36-3.48)^c^	1.20 × 10^−3^^c^

Abbreviations: ADHD, attention-deficit/hyperactivity disorder; AUDIT, The Alcohol Use Disorders Identification Test; BMI, body mass index; DAWBA, Development and Well-being Assessment; DEB, disordered eating behaviors; ESPAD, The European School Survey Project on Alcohol and Other Drugs; *ICD-10*, *International Statistical Classification of Disease, Tenth Revision*; OR, odds ratio; SDQ, Strengths and Difficulties Questionnaire.

^a^ All models controlled for sex and study site as covariates.

^b^ Analyses have been omitted based on nonsignificant results derived from generalized estimating equation models (eTables 3 and 4 in the Supplement).

^c^ Statistical significance with false discovery rate correction (corrected *P* < .05 for 54 tests). Benjamin-Hochberg threshold for significance of *P* = .03.

## Discussion

To our knowledge, this is the first study combining a large array of assessments, including genetics, personality, behavior, and mental health symptoms, with a longitudinal design to identify factors that were associated with the development of DEBs before their onset. Specifically, the finding that early ADHD symptoms, personality traits (low levels of agreeableness), alcohol and drug misuse, and self-harm were associated with the development of purging is novel. In addition, high levels of neuroticism, conduct problems, and self-harm predated binge eating, whereas high BMI was specifically associated with future dieting. Conduct problems as measured by the Strengths and Difficulties Questionnaire predated the development of obesity in adolescents, suggesting that these symptoms are associated with the development of ADHD,^46^ rather than being mere correlates of obesity in adolescence. In addition, our findings of distinct genetic predispositions, highlighting etiologic overlaps between BMI, neuroticism-related depressed affect, and ADHD with dieting, binge eating, and purging, respectively, were novel. The DEBs predated the onset of depression and anxiety symptoms, consistent with previous findings.^47^ Together, our findings suggest that genetic predispositions and psychopathologic processes associated with obesity, ADHD, and depression may be useful early and differential biomarkers of the vulnerability for eating disorders in adolescence.

Our findings that dieting was associated with development of both binge eating and purging, and that binge eating predates purging, but not vice versa, is unsurprising.^21^ Because we found that adolescents reporting dieting also had a higher BMI than their healthy counterparts, this finding can be placed in the context of the wide range of adverse outcomes known to be associated with high BMI in childhood or early adolescence.^48,49^

The traits found to predate the onset of binge eating, which include high levels of neuroticism (a predisposition toward emotionality, hypersensitivity, anxiety, worry, and depression), high rates of self-harm, and conduct problems, suggest that difficulty regulating emotions is an important characteristic of individuals at risk of developing binge-eating behaviors. Indeed, heightened emotionality in early childhood has been found to specifically predate binge eating, but not purging, at 14 and 16 years.^50^ For purging, low agreeableness and risk factors conceptually reflecting temperamental impulsivity, such as impulse-control disorders—ADHD and conduct problems—and addiction-related behaviors, were significant risk factors. The association between future purging behavior by ADHD symptoms might provide an explanation for the higher prevalence of ADHD symptoms in purging-associated phenotypes (ie, anorexia nervosa [AN] and bulimia nervosa binge eating/purging subtype compared with the AN restricting subtype).^28^ The associations between binge eating, purging, and conduct problems suggest a common underlying psychopathology associated with impulse control in binge-purge behavior. This is supported by previous neuroimaging findings in IMAGEN showing that failed inhibitory control is a risk factor for future development of binge eating or purging,^51^ and that have implicated shared neural circuitry, including the cognitive control system and the reward system, in the pathophysiology of ADHD and EDs.^52^ Overall, these findings suggest that developing early interventions focusing on emotion regulation and impulsivity, which has recently been proposed as a transdiagnostic treatment across anxiety, depression, substance misuse, and EDs,^53^ may be an effective strategy for prevention and treatment of EDs and comorbid disorders.

Although genetic factors are implicated in the etiology of EDs, research on the genetics of EDs has been limited and focused on AN.^54^ Our findings improve our understanding of risk for these disorders in several ways. The PRS for BMI, ADHD, and neuroticism differentially mediated associations between their respective phenotypes and DEBs, possibly accounted for by differences in variance explained by each PRS. Unsurprisingly,^21^ our data suggested that genetic predisposition for overweight or obesity, by increasing the likelihood for higher BMI, may facilitate the development of subsequent DEBs in adolescence and is significantly mediated by phenotypic BMI. Our findings of a shared genetic risk between ADHD and purging indicated the existence of shared pathophysiologic mechanisms between ADHD and EDs with purging phenotypes, as discussed above. As for neuroticism, the finding that genes specifically contributing to the depressed affect aspect of neuroticism were associated with binge eating suggested a shared etiology between binge eating and depressed affect. Depressed affect and worry, the 2 genetically homogeneous item clusters for neuroticism, share substantial yet sometimes opposite genetic correlations with other traits. Positive genetic correlations between depressed affect and childhood obesity and BMI contrast negative genetic correlations between the worry cluster and childhood obesity and BMI.^40^ However, although both depressed affect and worry have considerable genetic overlap with depression and anxiety disorders,^40^ their genetic correlations with AN (depressed affect) and ADHD (worry) were exclusive. This suggests that a shared set of genes predisposing to depressed affect, high BMI, and ADHD may contribute to observed comorbidities between these traits. By contrast, a distinct set of genes may predispose to worry, low BMI, and AN. These observations support well our findings of positive associations between BMI and ADHD and the development of binge-eating or purging behaviors.

### Strengths and Limitations

This study used a European-based population sample of more than 1600 adolescents, enabling multilevel longitudinal analyses of biopsychosocial measures. The major strengths of this study include its population-based setting with a large number of participants reporting DEBs and a comprehensive array of psychopathology and behaviors both preceding and as outcomes of DEBs. In addition, we targeted subclinical DEBs to make findings applicable to a large population and inform the design of targeted prevention programs.

There are several limitations to consider. First, the limited number of boys reporting DEBs did not enable us to investigate sex differences in the development of DEBs. Second, other factors not included in our analyses, such as socioeconomic status, are likely to have confounding effects. In addition, the internal consistency of several validated measures was lower than expected in this sample, which could bias our results. Finally, because this study measured participants aged 14 years for a baseline assessment, the developer groups could capture only individuals with the onset of disordered eating behaviors after age 14.

## Conclusions

This study found that genetic predispositions and psychopathological mechanisms related to obesity, ADHD, and personality were differentially associated with the vulnerability for eating disorders in adolescence. These results may have clinical implications for targeted ED prevention programs.

## Notes

Supplement. **eAppendix.** Materials and Methods **eReferences.** **eTable 1.** Prevalence of Disordered Eating Behaviors in the IMAGEN Sample Across 3 Time Points **eTable 2.** Prevalence of DEB Development in the IMAGEN Sample Between 16 and 19 Years in Those Reporting No DEBs at 14 Years **eTable 3.** Prevalence at Age 14 Years of Mental Health Disorders, Emotional and Behavioural Problems and Substance Use Behaviors in the IMAGEN Sample by DEB or HC Status **eTable 4.** Relationship Between DEBs, Symptoms of Mental Health Disorders and Personality Traits Across 3 Time Points in Adolescence: GEE Models **eTable 5.** Associations Between and Polygenetic Risk Scores (PRS) and DEBs at Different Ages **eTable 6.** PRS Summary Table With Optimal *P* Value Thresholds **eFigure.** Mediating Effect of BMI (a and b), ADHD Symptoms (c), and Neuroticism (d) Phenotype in the Relationship Between BMI, ADHD, and Neuroticism and DEBs
