# Supplementary material for: Association of Genetic and Phenotypic Assessments With Onset of Disordered Eating Behaviors and Comorbid Mental Health Problems Among Adolescents
Source: JAMA Netw Open. 2020 Dec 2;3(12):e2026874. doi: 10.1001/jamanetworkopen.2020.26874 (PMC7711322; doi:10.1001/jamanetworkopen.2020.26874)
Supplement: Supplement. — eAppendix. Materials and Methods eReferences. eTable 1. Prevalence of Disordered Eating Behaviors in the IMAGEN Sample Across 3 Time Points eTable 2. Prevalence of DEB Development in the IMAGEN Sample Between 16 and 19 Years in Those Reporting No DEBs at 14 Years eTable 3. Prevalence at Age 14 Years of Mental Health Disorders, Emotional and Behavioural Problems and Substance Use Behaviors in the IMAGEN Sample by DEB or HC Status eTable 4. Relationship Between DEBs, Symptoms of Mental Health Disorders and Personality Traits Across 3 Time Points in Adolescence: GEE Models eTable 5. Associations Between and Polygenetic Risk Scores (PRS) and DEBs at Different Ages eTable 6. PRS Summary Table With Optimal P Value Thresholds eFigure. Mediating Effect of BMI (a and b), ADHD Symptoms (c), and Neuroticism (d) Phenotype in the Relationship Between BMI, ADHD, and Neuroticism and DEBs [file jamanetwopen-e2026874-s001.pdf]

Supplementary Online Content

Robinson L, Zhang Z, Jia T, et al; IMAGEN Consortium. Association of genetic and phenotypic assessments with onset of disordered eating behaviors and comorbid mental health problems among adolescents. *JAMA Netw Open*. 2020;3(12):e2026874. doi:10.1001/jamanetworkopen.2020.26874

eAppendix. Materials and Methods
eReferences.
eTable 1. Prevalence of Disordered Eating Behaviors in the IMAGEN Sample Across 3 Time Points
eTable 2. Prevalence of DEB Development in the IMAGEN Sample Between 16 and 19 Years in Those Reporting No DEBs at 14 Years
eTable 3. Prevalence at Age 14 Years of Mental Health Disorders, Emotional and Behavioural Problems and Substance Use Behaviors in the IMAGEN Sample by DEB or HC Status
eTable 4. Relationship Between DEBs, Symptoms of Mental Health Disorders and Personality Traits Across 3 Time Points in Adolescence: GEE Models
eTable 5. Associations Between and Polygenetic Risk Scores (PRS) and DEBs at Different Ages
eTable 6. PRS Summary Table With Optimal *P* Value Thresholds
eFigure. Mediating Effect of BMI (a and b), ADHD Symptoms (c), and Neuroticism (d) Phenotype in the Relationship Between BMI, ADHD, and Neuroticism and DEBs

This supplementary material has been provided by the authors to give readers additional information about their work.

eAppendix.

Materials and Methods

Participants

The IMAGEN study is a population-based study conducted across 8 study sites in Europe. While the key recruitment criteria was age 14 years at baseline, two other criteria were used to maximize our study: (1) ethnic homogeneity to reduce stratification effects for genetic analyses, and (2) sample diversity in terms of socioeconomic status, academic achievement and behavioural/emotional functioning. Participants from the IMAGEN cohort[1] who reported ED behaviors in the DAWBA (Development and Wellbeing Assessment) were included from three waves (14+, 16+ and 19+). A total number of 2,463 individuals where recruited from high schools across Europe at the age of 14 years old (BL). The first follow-up assessment was done at the age of 16 years old (FU1) and a second follow-up assessment was conducted at the age of 19 years old (FU2). Full details of the procedures employed by the IMAGEN study, including details on ethics, recruitment, standardized instructions for administration of the psychometric and cognitive behavioural measures, and for blood collection and storage are available to view in the standard operating procedures for the IMAGEN project (http://www.imagen-europe.com/en/ Publications_and_SOP.php). Informed consent was obtained from all participants and their parents/guardians.

The final sample for this study included N=1,623 adolescents, who had participated in BL, FU1 or FU2 assessments. Attrition and drop-out from the study were predicted by gender, with girls more likely to participate in FU2 but not FU1 (OR: 0.86; 95% CI: 0.71 – 0.96). Missingness at FU1 was predicted by BL dieting (OR: 2.2; 95% CI: 1.13 – 2.15) and at FU2 was predicted by BL binge eating, purging and dieting (OR: 1.77; 95% CI: 1.02 – 2.27; OR: 1.89; 95% CI: 1.14 – 2.37; OR: 1.44; 95% CI: 1.13 – 2.01). Only individuals with complete cases were included in statistical analyses.

Assessments of ED psychopathology

Disordered Eating Behaviors: These were derived from adolescent self-report on the DAWBA section P (Eating Behaviors)[2] as follows:

Binge eating: *Responding* "Yes" to the following statement: '*sometimes people lose control over what they eat, and then they eat a very large amount of food in a short time. For example, they may open the fridge and eat as much as they can find – eating and eating until they feel*

© 2020 Robinson L *JAMA Network Open*.

physically ill. This usually happens when people are by themselves.' Participants were asked how many times they engaged in this behaviour over the past 3 months. Those responding once or more were included in this group.

Purging*: Responding* "Yes" to any of the following statements: '*Have you ever deliberately made yourself vomit (throw up)?' or ' Over the last three months, have you done any of the following to avoid putting on weight: vomit/ taking pills or medicines in order to lose weight?'.*

Dieting: *Responding* "A lot" to any of the following statements recording the frequency of their eating behaviour: *eating less at meals, skipping meals, going without food for long periods, e.g. all day or most of the day.*

Distress: distress about eating pattern and concern about body shape and weight was assessed at each time point by answers to the question: *'You have told me about your eating pattern and concern about weight or body shape. Are you 'Not at all', 'A little', 'A medium amount' or 'A great deal' distressed by this?* .

Eating Disorder Examination Questionnaire (EDEQ): We also used the EDEQ[3] to assess attitudinal aspects of ED (Restraint, Eating Concern, Weight Concern and Shape Concern) over the previous 28 days at age 19 years (i.e., at FU2).

Body mass index (BMI)

BMI (kg/m^2) at BL and FU2 was derived from objective height and weight measurements collected by the study researcher and transformed to age- and sex-adjusted z-scores based on the Centre for Disease Control and Prevention Growth Chart[4]. 'Underweight developer' and 'Overweight developer' groups were defined as individuals with a BMI z-score between 5th and 85th percentile at age 14 and above the 85th (overweight) or below the 5th (underweight) percentile by age 19.

Clinical, behavioral and personality-based assessments

Clinical assessments: DAWBA computer-based predictions scores[5] from self-reports were used to estimate probability of having a mental health disorder. Diagnoses investigated in this study were included if >5% of the IMAGEN sample scored above zero or 0.01% for the DAWBA computer prediction. These include Depression, Generalized Anxiety, Obsessive Compulsive Disorder (OCD), Conduct Disorder, Post Traumatic Stress Disorder (PTSD), Social Phobia, ADHD (Attention Deficit Hyperactivity Disorder), Oppositional Defiant Disorder, Separation Anxiety and Panic Disorder. Self-reports of lifetime history of Deliberate Self-Harm (DSH) was also investigated. Autism spectrum disorders, Agoraphobia, Tic

Disorders, Specific Phobias and Hyperkinesis computer prediction scores were excluded due to their low prevalence in this population. All DAWBA prediction scores were available at all three time-points, aside from ADHD and Separation Anxiety which were reported only at baseline and FU1 assessments. We derived computer-predicted DAWBA diagnoses by combining DAWBA scores 0-2 (probability of having the relevant diagnosis <1%) as 'healthy controls' and levels 3-5 (probability of having the diagnosis >15%) as 'cases'.

Development of mental health problems: We used longitudinal assessments of DAWBA diagnoses, as defined above, to identify groups of individuals likely to develop mental health disorders over time. We defined 'developers'' as individuals identified as a 'case' (e.g., with a predicted diagnosis of depression) at age 16 or 19, but not at baseline (14y).

*Addiction-related behaviors***:** These were assessed with two questionnaires, the European School Survey Project on Alcohol and Drugs (ESPAD[6]) and the Alcohol Use Disorders Identification Test (AUDIT[7]). The primary questions of interest regarding the ESPAD were lifetime binge drinking (> 3 occasions), drug use ever and smoking (past month). Binge drinking was a categorical variable based on reporting 3 or more episodes of drunkenness in the past year, versus no episodes of drunkenness (On how many occasions (if any) have you been drunk from drinking alcoholic beverages?). Drug-use was a categorical variable and defined as any drug intake in the past year. Smoking was also a categorical variable with 'cases' defined as those who have smoked in the past month, and 'controls' those who have never smoked. In the case of categorical variables, drug-use, binge drinking and smoking 'developer' groups were defined as individuals who were 'cases' at age 16 or 19, but not as baseline (14y). The AUDIT was used to assess alcohol dependence as a continuous variable using the total AUDIT score.

Longitudinal Trajectory analysis for personalities

We hypothesised that DEBs had an impact on how personalities change over time. To test this, the slopes of the trajectories for personalities were compared between the DEB developers and controls by using linear mixed-effect regression models, implemented in the lmer4 [36] and lmerTest [37] packages of the R software (v3.5.0).The age variable was coded as 0, 2 and 5 to reflect the gap between phases (14y, 16y and 19y). Both the fixed effect of the group (developers vs. controls) and the random effect of participant were modelled on the intercept and the slop of the trajectories, controlling for gender and sites. The effect of interest was the effect of group on the slope, namely, the group×age interaction.

© 2020 Robinson L *JAMA Network Open*.

Acquisition of Genetic Data

DNA purification and genotyping were performed by the Centre National de Génotypage in Paris. DNA was purified from whole blood samples (~10 ml) preserved in BD Vacutainer EDTA tubes (Becton, Dickinson and Company) using Gentra Puregene Blood Kit (QIAGEN) according to the manufacturer's instructions. Genotype information was collected at 582,982 markers using the Illumina HumanHap610 Genotyping BeadChip. Single nucleotide polymorphisms with call rates of < 98%, minor allele frequency < 2% or deviation from the Hardy-Weinberg equilibrium ($p \leq 1\times10^{-4}$) were excluded from the analyses. Individuals with an ambiguous sex code, excessive missing genotypes (failure rate > 2%), and outlying heterozygosity (heterozygosity rate 3 standard deviations from the mean) were also excluded. Identity-by-state similarity was used to estimate cryptic relatedness for individual using PLINK software[8]. Closely related individuals with identity-by-descent (IBD > 0.1875) were eliminated from the subsequent analysis. Population stratification for the genomewide association study was examined using multidimensional scaling (MDS) analysis with HapMap populations as reference groups. Individuals with divergent ancestry (from Utah residents with ancestry from northern and western Europe) were excluded through visual inspection of the first 2 components.

Polygenic Risk Scores calculation

Polygenic Risk Scores (PRS) were calculated in the target genotypes to be a sum of risk alleles across SNPs with the *p*-values below a given threshold, weighted by the effect sizes obtained from the GWAS summary statistics. PRSice software (v1.2.5)[9] was used to obtain the PRSs. Clumping was performed to remove linkage disequilibrium (LD) using default PRSice parameters, i.e., r^2 threshold=0.1, window size = 250kb. A regression model was performed to test the association between the PRSs and the target phenotype, involving sex, acquisition sites and population stratification (the first four MDS components of the genotype data) as covariates. Model fit (R^2) were estimated by subtracting the R^2 for the null model (involving the covariates only) from that for the full model. The *p*-value thresholds varied from 0.01 to 0.5 in a step of 0.01, in order to achieve the best model fit. PRSs were derived from the best-fit model.

We used mediation models to investigate whether neuroticism (BL), BMI (BL) and ADHD (SDQ hyperactivity FU1) phenotype mediated the relationship between PRS for neuroticism,

© 2020 Robinson L *JAMA Network Open*.

BMI and ADHD and DEBs. Control variables included sex, acquisition site and population stratification. The continuous variables were transformed to *z*-scores. Confidence intervals for the indirect effect were estimated with 5000 bootstrap samples by using the PROCESS macro (v3.2, http://processmacro.org) in SPSS (v25, IBM Corporation).

Statistical Analyses

Longitudinal analyses of predictors and outcomes of DEBs across adolescence: Generalised Estimating Equations (GEE) using DEBs as time-varying predictors and logistic and linear regression models were used to investigate the predictors, correlates and outcomes of DEBs. All continuous variables were standardised and their statistical output reported as z-scores. The odds of presenting with each of the outcomes were estimated using an unstructured working correlation structure and a robust estimation of standard errors. All analyses were adjusted for covariates including sex and study site in stepwise regression models.

eReferences

1. Schumann G, Loth E, Banaschewski T, et al. The IMAGEN study: reinforcement-related behaviour in normal brain function and psychopathology. *Mol Psychiatry*. 2010;15(12):1128.
2. Goodman A, Heiervang E, Collishaw S, Goodman R. The ‘DAWBA bands’ as an ordered-categorical measure of child mental health: description and validation in British and Norwegian samples. *Soc Psychiatry Psychiatr Epidemiol*. 2011;46(6):521-532.
3. Fairburn CG, Beglin SJ. Assessment of eating disorders: Interview or self-report questionnaire?. International journal of eating disorders. 1994 Dec;16(4):363-70.
4. Ogden CL, Kuczmarski RJ, Flegal KM, et al. Centers for Disease Control and Prevention 2000 growth charts for the United States: improvements to the 1977 National Center for Health Statistics version. *Pediatrics*. 2002;109(1):45-60.
5. Goodman A, Heiervang E, Collishaw S, Goodman R. The ‘DAWBA bands’ as an ordered-categorical measure of child mental health: description and validation in British and Norwegian samples. Social psychiatry and psychiatric epidemiology. 2011 Jun 1;46(6):521-32.
6. Hibell B, Guttormsson U, Ahlström S, et al. The 2007 ESPAD report. 2009;35:1-408.
7. Bush K, Kivlahan DR, McDonell MB, Fihn SD, Bradley KA. The AUDIT alcohol consumption questions (AUDIT-C): an effective brief screening test for problem drinking. *Arch Intern Med.*. 1998;158(16):1789-1795.
8. Purcell S, Neale B, Todd-Brown K, et al. PLINK: a tool set for whole-genome association and population-based linkage analyses. *Am J Hum Genet*2007;81(3):559-575.
9. Euesden J, Lewis CM, O’Reilly PF. PRSice: polygenic risk score software. *Bioinformatics (Oxford, England).* 2014;31(9):1466-1468.

© 2020 Robinson L *JAMA Network Open*.

eTable 1. Prevalence of Disordered Eating Behaviors in the IMAGEN Sample Across 3 Time Points

**Baseline**	**N=1509**	**No DEBs**	**DEBs N=307**		
			**Binge Eating**	**Purging**	**Dieting**
	N (% total sample)	1202 (79.65%)	107 (7.09%)	124 (8.21%)	160 (10.603%)
	% female	49.08%	**88.24%***	**77.97%***	**77.78%***
	BMI Mean (SD)	20.78 (3.12)	**22.66* (3.88)**	**22.47* (3.63)**	**22.71* (3.42)**
	BMI Mean Z-Score (SD)	0.19 (0.94)	**0.65 * (0.77)**	**0.63* (0.71)**	**0.71* (0.75)**
	% reporting Distress	N/A	**81.31%***	**84.84%***	**76.1%***
**FU1**	**N=1317**	**No DEBs**	**DEBs N=331**		
	N (% total sample)	986 (74.86%)	141 (10.71%)	177 (13.43%)	159 (12.07%)
	% female	49.98%	**84.33%***	**80.12%***	**78.43%***
	% reporting Distress	N/A	**85.1%***	**83.09%***	**87.42%***
**FU2**	**N=853**	**No DEBs**	**DEBs N=282**		
	N (% total sample)	571 (66.94%)	108 (12.66%)	178 (20.86%)	114 (13.36%)
	% female	50.59%	**80.39%***	**80.92%***	**77.06%***
	BMI Mean (SD)	22.01 (3.74)	**26.26* (5.12)**	**24.33* (4.46)**	**25.35* (3.66)**
	BMI Mean Z-Score (SD)	-0.14 (1.03)	**0.47* (0.95)**	**0.31* (0.89)**	**0.53* (0.88)**
	% reporting Distress	N/A	**83.03%***	**85.92%***	**83.33%***

** Indicates that differences between DEB groups and healthy controls are statistically significant to p<0.05 when controlling for covariates including gender and study site. Gender was not included as a covariate when gender differences were investigated. Binge-eating, purging and dieting are not mutually exclusive groups; thus, the total sample is not the sum of individual groups. Bonferroni correction applied to n=8 tests, with analyses of BMI and BMI z-scores considered as one test. P-value threshold = 0.0062.*

© 2020 Robinson L *JAMA Network Open*.

eTable 2. Prevalence of DEB Development in the IMAGEN Sample Between 16 and 19 Years in Those Reporting No DEBs at 14 Years

		**No DEBs**	**DEB Development N=322**		
			Binge Eating Development	Purging Development	Dieting Development
	N (% total sample)	922 (74.89%)	167 (13.56%)	207 (16.81%)	86 (7.06%)
	% female	47.15%	**74.85%***	**71.98%***	**65.12%***
	BL BMI Mean (SD)	20.47 (2.68)	**21.72* (3.45)**	**21.48* (3.08)**	**22.37* (3.54)**
	BL BMI Mean Z-Score (SD)	-0.12 (0.93)	**0.46* (0.85)**	**0.43* (0.79)**	**0.64* (0.78)**
**EDEQ Scores FU2 Mean (SD)**	Dietary Restraint	0.69 (1.11)	1.99 (1.61)	**1.66* (1.53)**	**2.28* (1.65)**
	Eating Concern	0.30 (0.63)	**1.55* (1.49)**	**1.15* (1.35)**	1.39 (1.51)
	Weight Concern	1.06 (1.31)	2.81 (1.75)	2.46 (1.82)	2.91 (1.85)
	Shape Concern	1.13 (1.24)`	2.75 (1.49)	2.46 (1.61)	**2.91* (1.56)**
	EDEQ Global	0.79 (0.96)	**2.28* (1.39)**	**1.93* (1.39)**	**2.37* (1.46)**

** Indicates that differences between DEB groups and healthy controls are statistically significant to p<0.05 when controlling for covariates including gender and study site.. Gender was not included as a covariate when gender differences were investigated. Dietary restraint, eating concern, weight concern and shape concern were included in the same linear regression model. EDEQ global score was investigated in isolation. Binge-eating, purging and dieting are not mutually exclusive groups, thus the total sample is not the sum of individual groups. Bonferroni correction applied to n=7 tests, with analyses of BMI and BMI z-scores considered as one test. P-value threshold = 0.0071.*

© 2020 Robinson L *JAMA Network Open*.

eTable 3. Prevalence at Age 14 Years of Mental Health Disorders, Emotional and Behavioural Problems and Substance Use Behaviors in the IMAGEN Sample by DEB or HC Status

		**Binge-eating (14y)** **N=107**	**Purging (14y)** **N=124**	**Dieting (14y)** **N=160**	**No DEBs N=2,079**
		N (%)	N (%)	N (%)	N (%)
**DAWBA**	Depression	35 (32.7)	43 (34.9)	42 (26.4)	94 (7.8)
	Generalised Anxiety	20 (24.7)	20 (20.0)	22 (17.5)	23 (2.1)
	Self-Harm	34 (31.7)	45 (36.5)	52 (32.7)	66 (5.4)
	OCD	2 (2.6)	2 (2.3)	7 (5.9)	3 (0.2)
	Social Phobia	13 (12.8)	9 (7.6)	16 (10.5)	17 (1.4)
	Panic Disorder	4 (4.3)	3 (2.6)	4 (2.7)	9 (0.7)
	PTSD	1 (1.1)	3 (2.8)	3 (2.1)	2 (0.1)
	Conduct Disorder	17 (26.9)	23 (30.7)	15 (16.7)	66 (7.8)
	ADHD	5 (6.5)	10 (12.2)	7 (5.9)	84 (9.5)
	Oppositional Defiant (ICD-10)	5 (6.8)	10 (14.3)	9 (9.0)	66 (7.8)
		M (SD)	M (SD)	M (SD)	M (SD)
**SDQ**	Emotional problems	4.3 (2.5)	3.9 (2.3)	3.7 (2.4)	2.1 (1.8)
	Conduct problems	2.6 (1.7)	2.6 (1.7)	2.5 (1.5)	2.0 (1.5)
	Hyperactivity/inattention	5 (2.2)	4.3 (2.3)	4.3 (2.2)	3.9 (2.2)
	Peers problems	2.5 (2.1)	2.4 (1.9)	2.1 (1.6)	1.7 (1.5)
	Prosocial	7.8 (1.5)	7.4 (1/6)	7.9 (1.5)	7.5 (1.7)
**AUDIT**	Alcohol Misuse	1.9 (2.9)	2.5 (3.4)	2.2 (3.0)	2.9 (2.2)
		N (%)	N (%)	N (%)	N (%)
**ESPAD**	Drug Use (>year)	9 (8.4)	17 (13.71)	21 (13.21)	93 (7.2)
	Smoking	4 (3.7)	7 (5.6)	12 (7.5)	30 (2.5)
	Binge Drinking *	13 (33.3)	19 (36.5)	13 (46.9)	87 (30.8)

© 2020 Robinson L *JAMA Network Open.*

eTable 4. Relationship Between DEBs, Symptoms of Mental Health Disorders and Personality Traits Across 3 Time Points in Adolescence: GEE Models

		**Binge-eating N=278**		**Purging N=334**		**Dieting N=256**	
		OR	*p*	OR	*p*	OR	*p*
	BMI	**1.07 (1.03; 1.12)**	**2.3E-04**	**1.08 (1.04; 1.12)**	**7.3E-05**	**1.24 (1.17; 1.31)**	**4.8E-14**
**DAWBA**	Depression	**5.86 (4.01; 8.57)**	**5.60E-20**	**3.84 (2.67; 5.52)**	**3.90E-13**	**4.64 (2.99; 7.19)**	**6.20E-12**
	Generalised Anxiety	**6.22 (3.91; 9.87)**	**9.10E-15**	**4.61 (2.86; 7.44)**	**3.50E-10**	**12.23 (6.32; 23.63)**	**1.00E-13**
	Self-Harm	**4.88 (3.35; 7.05)**	**6.70E-17**	**9.56 (6.46; 14.15)**	**1.30E-29**	**10.34 (6.39; 16.69)**	**1.30E-21**
	OCD	**4.41 (1.18l 16.39)**	**2.70E-02**	1.36 (0.29; 6.42)	6.93E-01	**12.15 (2.31; 64.03)**	**3.20E-03**
	Social Phobia	**4.48 (2.51; 8.01)**	**4.00E-07**	1.84 (1.003; 3.39)	4.90E-02	**5.09 (2.31; 11.17)**	**5.00E-05**
	Panic Disorder	**3.82 (1.14; 12.802)**	**3.00E-02**	**4.701 (1.16; 19.08)**	**3.00E-02**	1.99 (0.35; 11.54)	4.40E-01
	PTSD	**4.07 (1.57; 10.52)**	**3.70E-03**	**9.23 (3.35; 25.32)**	**1.60E-05**	**9.85 (2.51; 38.58)**	**1.00E-03**
	Conduct Disorder	**5.57 (3.07; 10.12)**	**1.60E-08**	**8.89 (4.86; 16.23)**	**1.20E-12**	**6.604 (3.201; 13.63)**	**3.30E-07**
	ADHD	1.96 (0.81; 4.78)	1.36E-01	**4.03 (1.67; 9.68)**	**1.80E-03**	2.44 (0.75; 7.91)	1.78E-01
	Oppositional Defiant (ICD-10)	**2.24 (1.09; 4.601)**	**2.70E-02**	**3.03 (1.42; 6.46)**	**3.90E-03**	2.65 (1.01; 6.98)	4.80E-02
**SDQ**	Emotional problems	**1.38 (1.29; 1.49)**	**8.40E-19**	**1.31 (1.22; 1.41)**	**1.00E-13**	**1.45 (1.33; 1.58)**	**3.80E-17**
	Conduct problems	**1.32 (1.19; 1.45)**	**5.70E-08**	**1.35 (1.22; 1.502)**	**3.60E-09**	**1.38 (1.22; 1.57)**	**1.60E-07**
	Hyperactivity/inattention	**1.28 (1.18; 1.38)**	**7.80E-11**	**1.13 (1.05; 1.21)**	**8.70E-04**	**1.13 (1.04; 1.23)**	**3.40E-03**
	Peers problems	**1.28 (1.16; 1.41)**	**2.00E-07**	**1.209 (1.09; 1.33)**	**1.20E-04**	**1.31 (1.16; 1.46)**	**3.30E-06**
	Prosocial	**0.89 (0.81; 0.93)**	**3.80E-02**	1.01 (0.91; 1.12)	8.67E-01	1.01 (0.89; 1.13)	9.29E-01
**AUDIT**	Alcohol Misuse	**1.14 (1.08; 1.21)**	**1.80E-06**	**1.28 (1.21; 1.37)**	**4.50E-15**	**1.18 (1.11; 1.27)**	**8.80E-07**
**ESPAD**	Drug Use (>year)	**2.71 (1.71; 4.29)**	**2.20E-05**	**5.32 (3.48; 8.12)**	**9.90E-15**	**1.92 (1.13; 3.28)**	**1.70E-02**
	Smoking	**2.74 (1.65; 4.54)**	**8.60E-05**	**4.61 (2.82; 7.54)**	**1.10E-09**	**2.76 (1.51; 5.01)**	**8.70E-04**
	Binge Drinking *	**0.98 (0.72; 1.35)**	9.38E-01	**1.75 (1.25; 2.43)**	**9.20E-04**	1.21 (0.85; 1.75)	2.76E-01
**NEO-FFI**	Neuroticism	**2.37 (1.98; 2.83)**	**3.30E-21**	**2.08 (1.74; 2.48)**	**2.40E-16**	**2.47 (2.03; 3.01)**	**2.30E-19**

© 2020 Robinson L *JAMA Network Open*.

	Extraversion	**0.77 (0.66; 0.91)**	**2.40E-03**	**0.77 (0.66; 0.91)**	**2.10E-03**	**0.79 (0.66; 0.96)**	**1.60E-02**
	Openness	1.17 (0.97; 1.41)	1.04E-01	1.17 (0.98; 1.38)	6.70E-02	0.89 (0.73; 1.08)	2.55E-01
	Agreeableness	**0.61 (0.51; 0.79)**	**8.80E-09**	**0.65 (0.55; 0.76)**	**1.30E-07**	**0.65 (0.55; 0.79)**	**4.60E-06**
	Conscientiousness	0.67 (0.45; 1.01)	5.60E-02	**0.801 (0.66; 0.98)**	**2.80E-02**	0.94 (0.77; 1.13)	4.82E-01

All models controlled for gender and study site as covariates.* ***Bold* *indicates statistical significance with FDR correction (FDR<0.05 for 72 tests). Benjamin Hochberg p-value threshold for significance = 0.038*

© 2020 Robinson L *JAMA Network Open*.

eTable 5. Associations Between and Polygenetic Risk Scores (PRS) and DEBs at Different Ages
Odds Ratios are presented with the lower bound of the 95% confidence interval (CI) and *p*-values for one-tailed tests in parentheses.

Age	BMI PRS – Dieting OR (CI lower bound, *p*)	BMI PRS – Purging OR (CI lower bound, *p*)	ADHD PRS – Purging OR (CI lower bound, *p*)	Neuroticism PRS – Binge-eating		
				Neuroticism full scale PRS OR (CI lower bound, *p*)	'Depressed affect' PRS OR (CI lower bound, *p*)	'Worry' PRS OR (CI lower bound, *p*)
14y	**1.27 (1.08, 9.1E-03)**	**1.34 (1.12, 3.3E-03)**	1.21 (1,02, 0.034)	**1.32 (1.11 4.4E-03)**	**1.34 (1.12, 3.7E-03)**	1.20 (1.01, 0.044)
16y	**1.38 (1.17, 7.9E-04)**	**1.32 (1.14, 1.1E-03)**	**1.25 (1.08, 5.7E-03)**	**1.24 (1.06, 0.015)**	**1.29 (1.09, 6.7E-03)**	1.11 (0.94, 0.15)
19y	1.07 (0.089, 0.27)	1.17 (1.01, 0.044)	**1.23 (1.06, 0.012)**	1.21 (1.01, 0.042)	1.01 (0.84, 0.45)	1.15 (0.96, 0.10)

Logistic regression models were used, controlling for gender, study site and population stratification (the first four multidimensional scaling components of the genotype data). ***Bold*** *indicates statistical significance after Bonferroni correction of 3 tests (p-value threshold 0.017).*

© 2020 Robinson L *JAMA Network Open*.

eTable 6. PRS Summary Table With Optimal *P* Value Thresholds

	**optimal p threhold**	**variance explained**	**#SNP included**
**BMI**	0.25	5.15%	29995
**ADHD**	0.37	1.11%	49077
**Neuroticism**	0.09	1.64%	24879
**Depressed affect**	0.22	0.86%	40842
**Worry**	0.04	0.17%	14294

© 2020 Robinson L *JAMA Network Open*.

eFigure. Mediating Effect of BMI (a and b), ADHD Symptoms (c), and Neuroticism (d) Phenotype in the Relationship Between BMI, ADHD, and Neuroticism and DEBs

Control variables included sex, acquisition site and population stratification. PRS, polygenetic risk scores. Hyper = Hyperactivity/Inattention. *P<0.05, **P<0.01, ***P<0.001

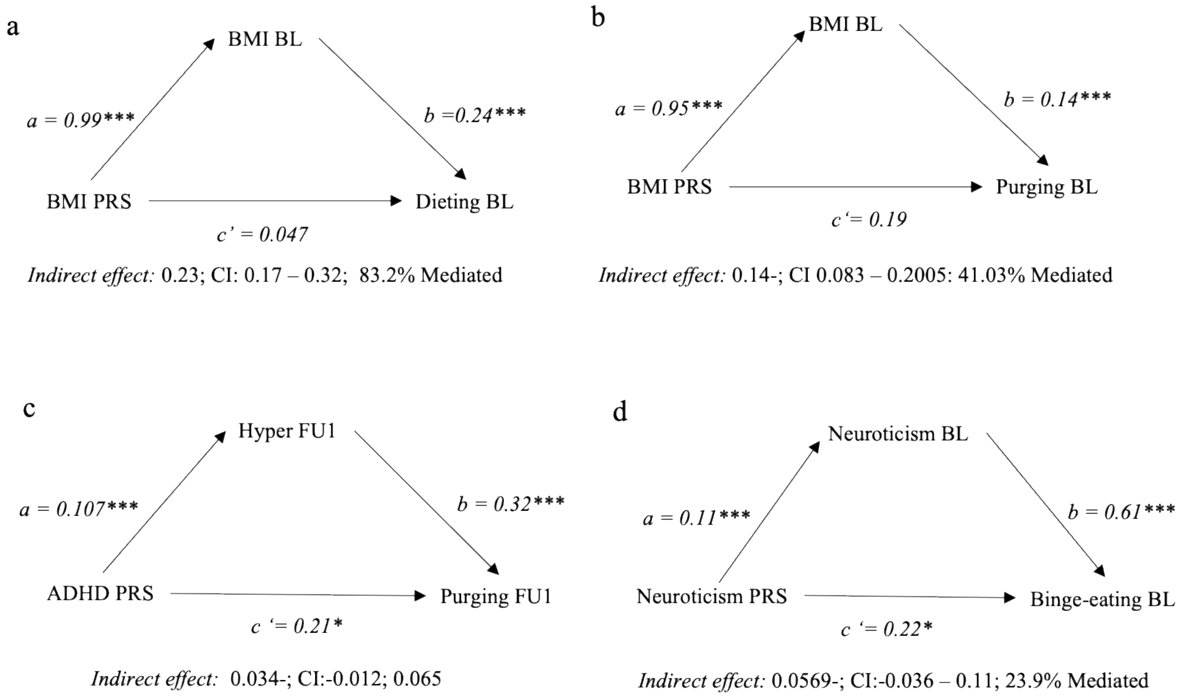

© 2020 Robinson L *JAMA Network Open*.
